# Advancements in Performance Optimization of Electrospun Polyethylene Oxide-Based Solid-State Electrolytes for Lithium-Ion Batteries

**DOI:** 10.3390/polym15183727

**Published:** 2023-09-11

**Authors:** Xiuhong Li, Yichen Deng, Kai Li, Zhiyong Yang, Xinyu Hu, Yong Liu, Zheng Zhang

**Affiliations:** 1 School of Mechanical Engineering, Hubei University of Technology, Wuhan 430000, China; 20200005@hbut.edu.cn (X.L.); 102110078@hbut.edu.cn (Y.D.); 102210149@hbut.edu.cn (K.L.); 20161024@hbut.edu.cn (Z.Y.); 19991012@hbut.edu.cn (X.H.); 2 School of Materials Science and Engineering, Beijing University of Chemical Technology, Chaoyang District, Beijing 100000, China

**Keywords:** electrospinning, PEO-based solid-state electrolyte, performance optimization

## Abstract

Polyethylene oxide (PEO)-based solid-state electrolytes for lithium-ion batteries have garnered significant interest due to their enhanced potential window, high energy density, and improved safety features. However, the issues such as low ionic conductivity at ambient temperature, substantial ionic conductivity fluctuations with temperature changes, and inadequate electrolyte interfacial compatibility hinder their widespread applications. Electrospinning is a popular approach for fabricating solid-state electrolytes owing to its superior advantages of adjustable component constitution and the unique internal fiber structure of the resultant electrolytes. Thus, this technique has been extensively adopted in related studies. This review provides an overview of recent advancements in optimizing the performance of PEO solid-state electrolytes via electrospinning technology. Initially, the impacts of different lithium salts and their concentrations on the performance of electrospun PEO-based solid-state electrolytes were compared. Subsequently, research pertaining to the effects of various additives on these electrolytes was reviewed. Furthermore, investigations concerning the enhancement of electrospun solid-state electrolytes via modifications of PEO molecular chains are herein detailed, and lastly, the prevalent challenges and future directions of PEO-based solid-state electrolytes for lithium-ion batteries are summarized.

## 1. Introduction

**Figure 1:**
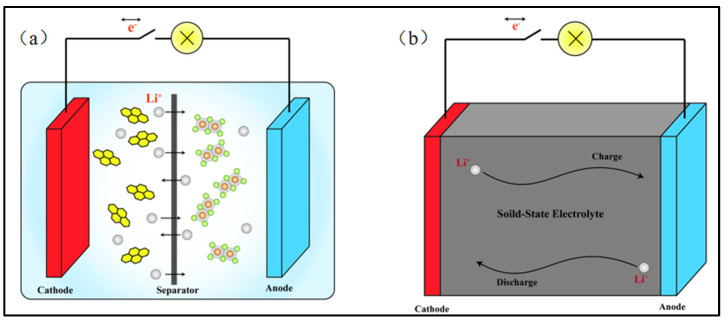
(**a**) Schematic diagram of lithium-ion battery structure. (Adapted with permission from [7]. Copyright 2013, American Chemical Society.) (**b**) Schematic diagram of lithium-ion solid-state battery.

The escalating energy crisis and environmental issue necessitate the developments of eco-friendly and sustainable energy alternatives [1]. Among diverse sustainable energy sources, electrochemical energy plays a pivotal role in the transition of fossil fuels to clean and renewable energies owing to its cost effectiveness and green characteristics. The predominantly utilized electrochemical energies include lead-acid, nickel-cadmium, and lithium-ion batteries. The latter has been widely employed in electronic products due to its superior operating voltage, energy density, charging and discharging rates, and adequate cycling stability [2,3]. Figure 1a depicts the core components of a traditional lithium-ion battery, which mainly consists of an anode, a cathode, an electrolyte, and a separator [4]. During operation, lithium ions move back and forth through the electrolyte and the separator to complete charging and discharging. Thus, the performance of the battery primarily depends on the properties of the electrolyte and the separator. Although most existing commercial lithium-ion batteries have excellent electrochemical properties, there are persisting concerns about the easy flammability of the electrolyte and the relatively poor mechanical property of the separator [5]. These drawbacks may lead to the electrolyte leakage, internal short circuiting, or even explosive accidents during a prolonged use or under an external force, thereby impeding their further applications [6].

To mitigate these issues and promote the development of lithium-ion batteries, researchers have proposed substituting the conventional electrolyte and separator with mechanically robust solid-state electrolytes, as shown in Figure 1b. Solid-state electrolytes present advantages such as good flexibility and electrode wettability, which avoid the use of liquid electrolytes and thus improve the stability, safety, and lifespan of lithium-ion batteries [8,9,10]. Additionally, the employments of solid-state electrolytes in lithium-ion batteries not only make them compact and lightweight but also offer a high energy density, thus extraordinarily enhancing their future application prospects [9,11].

Generally, solid-state electrolytes are primarily classified into two types: inorganic ceramics and organic polymers. Inorganic ceramic-based solid-state electrolytes possess superior electrical conductivity, yet their inadequate mechanical properties and high interface impedance with the electrode limit their use in solid-state electrolytes. In contrast, although the electrochemical properties of organic polymer-based solid-state electrolytes are inferior to those of inorganic ceramic-based solid-state electrolytes, their excellent plasticity and extraordinary mechanical properties make them preferred choices for solid-state electrolytes in lithium-ion batteries.

**Figure 2:**
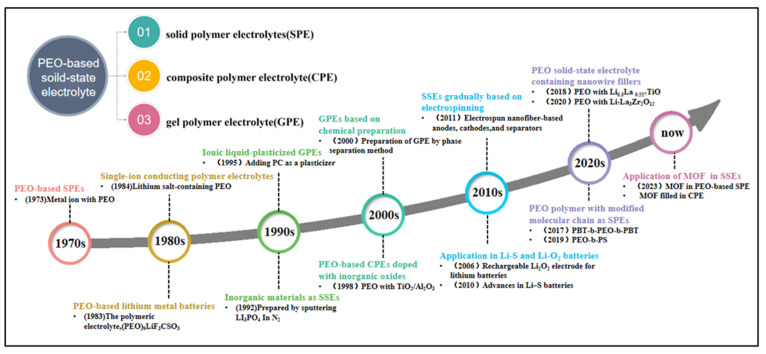
Development History of PEO Solid-State Electrolytes [23,26,27,28,29,30,31,32,33,34,35,36,37,38,39].

Commonly, the most used materials for organic polymer-based solid-state electrolytes of lithium-ion batteries include polymethyl methacrylate (PMMA) [12], polyacrylonitrile (PAN) [13], polyvinylidene difluoride (PVDF) [14], PEO [15], etc. Lots of studies have demonstrated that the obtained solid-state electrolytes made of different organic polymers exhibit diverse physiochemical features such as thermal stability, cell capacity, and mechanical strength [16,17]. For example, solid-state electrolytes prepared with PVDF show good mechanical properties and a wide electrochemical window. However, the relatively regular molecular structure and lower ionic conductivity of PVDF limit its further developments [10,18]. Conversely, PEO has emerged as one of the most promising materials for solid-state electrolytes in lithium-ion batteries because of its large dielectric constant and high ionic conductivity and the excellent solubility of lithium salts [19,20]. Furthermore, PEO is able to stabilize the interface between the solid-state electrolyte and the anode, thereby suppressing the growth of lithium dendrites and improving the cycling performance of the lithium-ion battery [21,22]. Though the research concerning PEO as an alternative of solid-state electrolyte began in 1973 [23], it has been implemented in a multitude of solid-state electrolytes, with its developmental timeline illustrated in Figure 2. In order to optimize the performance of lithium-ion batteries, current efforts primarily focus on three aspects: electrodes, solid-state electrolytes, and the overall structure of the batteries [24]. In terms of the composition of solid-state electrolytes in lithium-ion batteries, PEO exhibits extraordinary merits. However, being a semi-crystalline polymer, its lithium-ion transport rate at ambient temperature is relatively low, resulting in a low ionic conductivity for PEO-based solid-state electrolytes [25]. Consequently, many current studies focus on reducing the crystallinity of PEO to enhance the lithium-ion mobility of PEO-based solid-state electrolytes for lithium-ion batteries at room temperature.

Current methodologies for preparing solid-state electrolytes include casting, hot pressing, and electrospinning. Among them, the solution casting method is simple and low-cost and easily adjusts the thickness of the film [8]. The hot-pressing method is suitable for obtaining uniformly thick films, reducing the interfacial resistance of the solid-state electrolyte [40,41]. However, both methods have limitations in obtaining solid-state electrolytes with complex internal structures. In comparison, electrospinning not only allows for the fabrication of solid-state electrolytes with diverse internal structures but also offers benefits such as simplicity and cost-effectiveness. And this approach possesses the ability to manipulate the material composition to yield products with multiple functions, attracting much attention. This technique employs high-voltage electrostatic forces to stretch and solidify charged polymer solutions or melts, thus forming micro/nanofibers [42,43,44]. In contrast to the former two approaches, electrospinning provides customization of the fibers’ internal morphology by altering process conditions and polymer characteristics, leading to the generation of fibrous materials with unique structures such as core-shell, hollow, and porous. The resultant electrospun micro/nanofibers typically exhibit high porosity and specific surface area [45]. When these micro/nanofibers are employed as primary elements for solid-state electrolytes, they are able to improve internal lithium-ion mobility, thereby increasing the ionic conductivity of the obtained PEO-based solid-state electrolytes for lithium-ion batteries [46]. Moreover, these electrospun micro/nanofibers with great continuities allow for tight stackings, thus extending lithium-ion transport channels. This characteristic can not only alleviate the uneven lithium-ion deposition during battery usage but also render batteries with great mechanical strength and lower internal crystallinity of the electrospun polymer-based solid-state electrolytes. Therefore, the integration of electrospinning with other technologies has emerged as a primary approach for fabricating polymer-based solid-state electrolytes for lithium-ion batteries.

**Figure 3:**
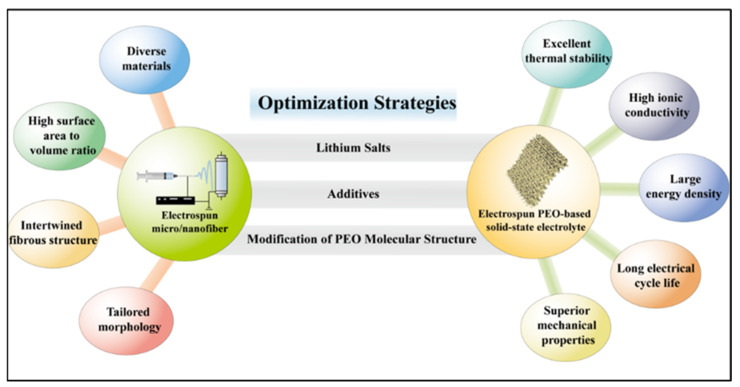
Optimization strategies for improving performance of PEO-based solid electrolyte based on electrospinning technology.

In summary, the introduction of electrospinning in the production of PEO-based solid-state electrolytes of lithium-ion batteries leverages the advantages of both PEO as a solid-state electrolyte material and the unique properties of electrospun micro/nanofibers. Accordingly, an increasing number of researchers are devoting their efforts toward studies related to electrospun PEO-based lithium-ion solid-state electrolytes. Presently, these explorations predominantly concentrate on enhancing the electrochemical, mechanical, and thermal performances of electrospun PEO-based lithium-ion solid-state electrolytes. Although noticeable advancements have been accomplished in recent years, the commercialization of electrospun PEO-based lithium-ion solid-state electrolytes demands a further refinement. In this review, a variety of strategies for performance improvement of electrospun PEO-based solid-state electrolytes have been concluded (Figure 3). Firstly, in this study the effects of concentrations and kinds of chosen lithium salts on the capabilities of electrospun PEO-based solid-state electrolytes are analyzed, offering a selection foundation of lithium salts for them. Then, the incorporation of appropriate additives to facilitate for improving the performance of electrospun PEO-based solid-state electrolytes is examined. Next, the refining of the PEO molecular chains to realize superior electrochemical abilities of electrospun PEO-based solid-state electrolytes is demonstrated. Finally, a conclusion and prospects of electrospun PEO-based solid-state electrolytes for lithium-ion batteries is provided.

## 2. Enhancement Strategies for Electrospun PEO-Based Solid-State Electrolytes

The efficiency of electrospun PEO-based solid-state electrolytes for lithium-ion batteries is mainly dependent on their electrochemical characteristics, mechanical properties, and thermal stability. Among them, electrochemical performance is paramount, which is determined by several significant factors such as lithium-ion conductivity, lithium-ion transfer number, and electrochemical stability window. Currently, there exists a considerable disparity between the electrochemical performance of solid-state electrolytes and conventional liquid electrolytes, which poses a significant barrier to further advancements of PEO-based solid-state electrolytes.

The ionic conductivity, a critical indicator in evaluating electrochemical performance, represents the transport rate of charged ions within a material. The low conductivity confines the battery to operate at a relatively small charge/discharge rate, thereby making it challenging to satisfy the operational requirements of standard devices. Plus, the semi-crystalline structure of PEO results in a high degree of crystallinity inside itself at room temperature, characterized by an abundance of internal crystalline regions. Direct usage of PEO as a solid-state electrolyte base for lithium-ion batteries would severely inhibit lithium-ion transportation within and between PEO chain segments owing to these crystalline regions. Consequently, these would lead to inefficient lithium-ion transport, thereby decreasing the ionic conductivity of PEO-based solid-state electrolytes (<10^−6^ S/cm at 25 °C).

**Figure 4:**
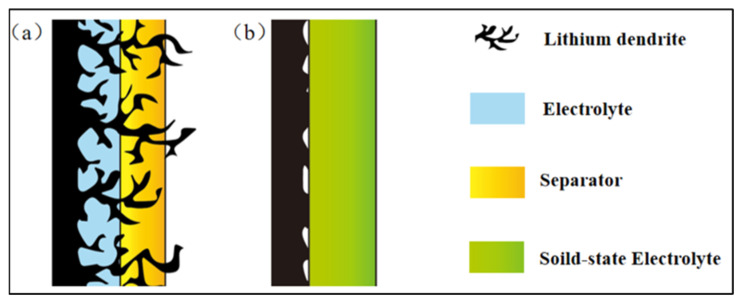
Comparison of dendrite growth in two different electrolyte systems: (**a**) liquid electrolyte system and (**b**) solid-state electrolyte system. (Adapted with permission from [50]. Copyright 2016, Wiley).

Additionally, lithium ions are gradually deposited on the electrode surface during lithium-ion battery operation, reduced to lithium monomers, and then form nucleated protrusions [47]. These protrusions diffuse into pores and defects on the electrolyte surface, inducing the formation of lithium dendrites. Lithium dendrites pose the risk of piercing the electrospun PEO-based solid-state electrolyte, triggering safety incidents [48,49]. Thus, an electrospun PEO-based solid-state electrolyte with robust mechanical properties is beneficial for hindering the growth of lithium dendrites, thereby enhancing safety and prolonging the lifespan of lithium-ion batteries (Figure 4).

Lastly, the thermal characteristics of the electrospun PEO-based solid-state electrolyte also substantially determine the safety of lithium-ion batteries. Since the operating temperature of the lithium-ion batteries inevitably rises to a certain extent for a long-time use, it would potentially cause the solid-state electrolyte to contract and deform, which would bring safety concerns regarding the lithium-ion batteries. Thus, the electrospun PEO-based solid-state electrolyte must maintain an appropriate thermal closure temperature to prevent short circuits or explosions due to excessive working temperature. When the lithium-ion batteries are in overheating scenarios, the solid-state electrolyte closes the ion transport channels, stopping the battery from continuing operation and avoiding hazardous accidents [51].

All in all, it is significant to take the above-mentioned factors into considerations to obtain a qualified electrospun PEO-based solid-state electrolytes. Commonly, the principal components of electrospun PEO-based solid-state electrolytes include lithium salts, additives, and the polymer matrix. Their most substantial performance optimization strategies rely on the three elements. Subsequently, the paper separately discusses the three different improvement avenues for performance optimizations and their accompanying specific impacts in terms of the above influencing parameters on the electrochemical capabilities of electrospun PEO-based solid-state electrolytes.

### 2.1. Lithium Salts for Electrospun PEO-Based Solid-State Electrolytes

**Figure 5:**
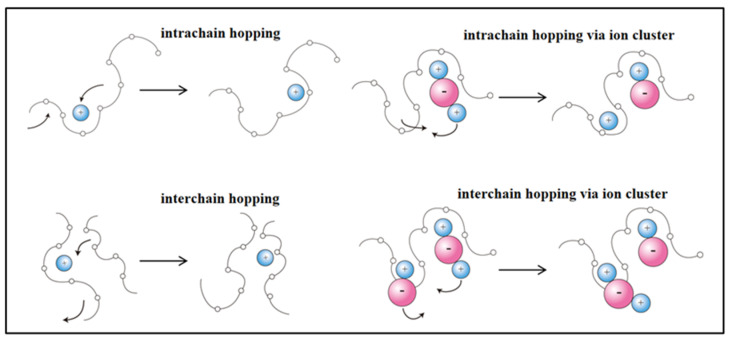
Mechanism of ion transport in PEO. (Adapted with permission from [59]. Copyright 2015, Royal Society of Chemistry.).

As a key component of solid-state electrolyte in lithium-ion batteries, lithium salts provide charge carriers to ensure normal functions of the batteries during operation; Figure 5 illustrates the transport mechanism of lithium ions in the PEO matrix [52,53]. To enhance the dissociation degree of the lithium salt anion in the solid-state electrolyte for supplying more charge carriers, the lithium salts utilized typically possess the following attributes: a large anion size, a low lattice energy, and a strong electrochemical stability. Furthermore, the applied lithium salts should enable the polymer matrix material of the solid-state electrolyte to exhibit an amorphous state. Presently, commonly employed lithium salts include lithium hexafluorophosphate (LiPF_6_), lithium perchlorate (LiClO_4_), lithium tetrafluoroborate (LiBF_4_), lithium chloride (LiCl), and lithium bis(trifluoromethanesulfonyl)imide (LiTFSI) [54]. The performance of the assembled solid-state electrolyte is influenced by both the concentration and type of these lithium salts.

The concentration of the used lithium salt impacts the ionic conductivity of electrospun PEO-based solid-state electrolytes. Generally, an increase in lithium salt concentration reduces the crystallinity of electrospun PEO-based solid-state electrolytes and elevates the lithium-ion migration number within them. However, an overlarge concentration can lead to the aggregation of the lithium salt, thereby impairing the lithium-ion migration efficiency [55]. Plus, the concentration of the lithium salt also affects the electrospinning process, further influencing the diameter and internal crystallinity of electrospun PEO micro/nanofibers. Hence, doping lithium salts at an appropriate concentration yields electrospun PEO micro/nanofibers of smaller diameters and improved molecular chain orientations. This can endow electrospun PEO-based solid-state electrolytes with a higher porosity and a smaller crystalline region [56,57]. Consequently, the migration rate and ionic conductivity of lithium ions in the cell increase, producing a solid-state electrolyte with a superior performance [58].

For instance, Yalcinkaya et al. [57] demonstrated that the addition of lithium salts enlarges the diameter of electrospun micro/nanofibers. However, a study by Banitaba et al. [60] suggested that this might result from the incomplete volatilization of the solvent during the electrospinning process. Their findings indicate that adding lithium salt intensifies the electric field force on the electrospun jets, leading to the formation of finer micro/nanofibers. when the concentration of lithium salt exceeds 0.5 wt%, the strong electric field force will enlarge the electrospun jet flow and cause the fibers to thicken accordingly. Additionally, when the concentration exceeds approximately 2 wt%, the excess charges in the electrospun micro/nanofibers accumulate and generate a repulsive effect on the collection device, rendering it challenging to obtain the final fiber product. Additionally, a larger lithium salt concentration may deteriorate the mechanical properties of the solid-state electrolyte, which in turn affects the safety of the obtained lithium-ion batteries [61].

The type of the selected lithium salt in the electrospun PEO-based solid-state electrolyte also considerably influences its operational performance. Banitaba et al. [60] conducted experimental research on the influence of different lithium salts on the performance of electrospun PEO-based solid-state electrolytes. They prepared PEO-based solid-state electrolytes with varying concentrations of LiCl, LiTFSI, and LiClO_4_ for comparison. Compared to LiCl, the introduction of LiTFSI and LiClO_4_ increased the dielectric constant of the PEO polymer solution, thus enhancing the ionic conductivity of the electrospun PEO-based solid-state electrolyte. When the concentration of LiClO_4_ was 1.5 wt%, the ionic conductivity of the fabricated electrospun PEO-based solid-state electrolytes reached 3.3 × 10^−4^ S/cm at a room temperature, which was nearly an order of magnitude higher than that of solid-state electrolytes with other lithium salts. Similarly, Chang et al. [62] also used LiClO_4_ as a lithium salt, which led to a decrease in the crystallinity within the electrospun PEO fibers and consequently resulted in a solid-state electrolyte with a superior conductivity.

**Figure 6:**
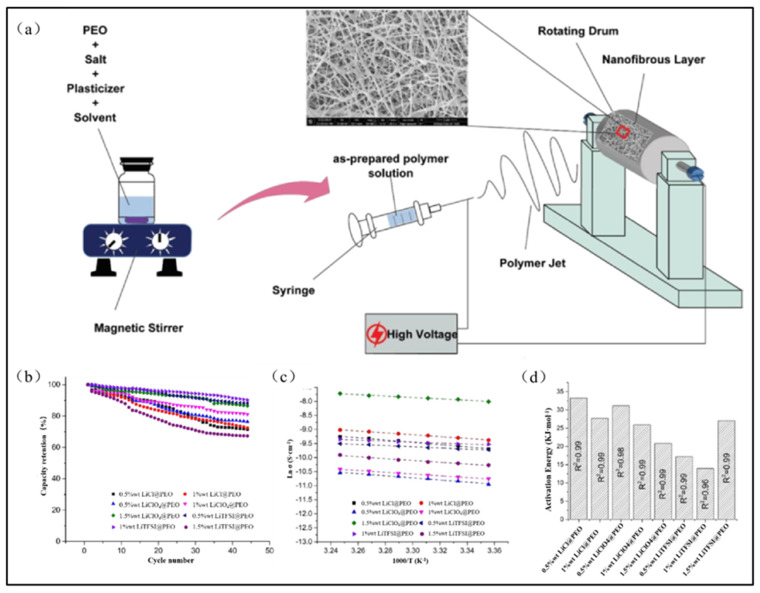
(**a**) Schematic illustration of electrospinning apparatus; (**b**) cycle stability of the as-spun membranes; (**c**) variation of conductivity with temperature; (**d**) activation energy of prepared solid-state electrolyte. (Reprinted with permission from [60]. Copyright 2020, Springer Nature.).

Additionally, different kinds of lithium salts exhibit varying lattice energy levels, which can affect their degree of dissociation in electrospun micro/nanofibers and the electrochemical properties of the solid-state electrolytes. For example, LiTFSI shows a lower lattice energy and dissociates more readily in electrospun micro/nanofibers compared with other lithium salts. Moreover, LiTFSI possesses larger anions, which can enhance the orientation of PEO molecular chains and decrease their crystallinity. The experimental results by Banitaba et al. [60] demonstrate that although LiTFSI exhibits a slightly lower enhancement in terms of ionic conductivity compared to LiClO_4_ (Figure 6b), solid-state electrolytes prepared using LiTFSI in electrospun PEO exhibit higher cycling stability (Figure 6c). Walke et al. [63] added LiTFSI and LiBF_4_ to PEO to produce a PEO-based solid-state electrolyte via electrospinning. They found that the incorporation of LiTFSI effectively suppressed the crystallinity of PEO-based solid-state electrolytes. In contrast to LiBF_4_, the electrospun PEO-based solid-state electrolyte containing LiTFSI exhibited a higher ionic conductivity and superior cycling stability.

Due to the favorable performance of LiClO_4_ and LiTFSI in PEO-based solid-state electrolytes, they are commonly employed in most of the current research on electrospun PEO-based solid-state electrolytes. Additionally, the concentration of lithium salts is a crucial factor affecting the performance of PEO-based solid-state electrolytes. Since the concentration of the lithium salt impacts the morphology of electrospun fibers, the ionic conductivity of the solid electrolyte initially increases and then decreases as lithium salt concentration gradually rises. Simultaneously, mechanical properties gradually deteriorate. Therefore, to ensure both the ion conductivity and the mechanical performance of PEO-based solid-state electrolytes, it is essential to adjust the concentration of lithium salts within an appropriate range.

### 2.2. Additives for Electrospun PEO-Based Solid-State Electrolytes

Optimizing the parameters of lithium salts alone cannot fully improve the electrochemical properties of electrospun PEO-based solid-state electrolytes. Integrating additives into the polymer matrix can improve not only the electrochemical characteristics but also the mechanical features of electrospun PEO-based solid-state electrolytes. Currently, common additives are divided into three primary categories: plasticizers [64,65,66,67], inorganic nanofillers [68,69,70], and other polymers [71].

#### 2.2.1. Plasticizers

Plasticizer molecules can enter polymer molecular chains, mitigating the interactions between these chains and preventing crystallization. Hence, the introduction of plasticizers to PEO-based solid-state electrolytes can reduce their glass transition temperature and crystallinity. Moreover, they can also promote the dissociation of lithium salts in PEO polymers and increase the number of free ions within. These can optimize the transport efficiency of lithium ions within the material [72,73], thereby enhancing the ionic conductivity of PEO-based solid-state electrolytes. Normally, common plasticizers include dimethyl carbonate (DMC), dioctyl adipate (DOA), dibutyl phthalate (DBP), diethyl carbonate (DEC), propylene carbonate (PC), and ethylene carbonate (EC).

**Figure 7:**
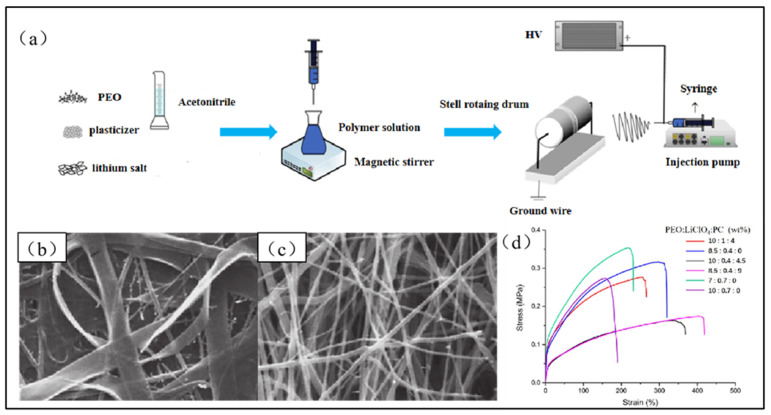
(**a**) Electrospinning apparatus used for the fabrication of the electrospun electrolytes. (Reprinted with permission from [76]. Copyright 2020, Elsevier B.V.) (**b**,**c**) SEM images of electrospun fibers with and without plasticizer. (**d**) Typical tensile stress–strain curves of several nanofibrous electrolytes. (Reprinted with permission from [74]. Copyright 2019, Wiley.).

Banitaba et al. [74] investigated electrospun micro/nanofibers of pure PEO and PEO incorporated with a PC plasticizer for preparing solid-state electrolytes, as shown in Figure 7a. The inclusion of a PC plasticizer reduces the viscosity of the polymer solution, thereby facilitating the elongation of micro/nanofibers during the electrospinning. This process leads to a notably smaller fiber diameter and a more uniform circular cross-section, both of which are beneficial for improving the electrochemical properties of electrospun micro/nanofibers (Figure 7b,c). The ionic conductivity of the resulting electrospun fibers supplemented with PC plasticizers was found to be nearly three times higher than that of pure PEO fibers. Although plasticizers serve to enhance the electrochemical abilities of electrospun PEO-based solid-state electrolytes, they also diminish the tensile strength of the electrospun micro/nanofibers to a degree, leading to a decline in their mechanical strength (Figure 7d). Moreover, the addition of excessive plasticizers can impede the electrospinning process due to the low solution viscosity. Consequently, the amount of the plasticizer must be appropriately controlled to obtain electrospun PEO-based solid-state electrolytes with excellent electrochemical and mechanical properties.

The type of plasticizer also influences the performance of electrospun PEO-based solid-state electrolytes. The effects of plasticizers on solid-state electrolytes depend on their relative permittivity (ε), viscosity, and interaction with lithium salts. PC (ε = 64.4) and EC (ε = 89) are frequently used in electrospun PEO-based solid-state electrolytes due to their high relative permittivity. The specific binding energy of EC with lithium ions is superior to that of PC. Therefore, EC exerts a more significant influence on the ionic conductivity of solid-state electrolytes than PC [64,75]. Another study by Banitaba et al. [76] evaluated the effects of a mixture of EC and PC plasticizers on the performance of electrospun PEO-based solid-state electrolytes. They found that an overly high PC concentration decreased the cyclic stability of the resulting solid-state electrolytes. The ionic conductivity of the resultant solid-state electrolytes peaked when the EC:PC ratio was 3:1. Besides the commonly used plasticizers, succinonitrile (SN) has emerged as a popular plasticizer to enhance the electrochemical properties of solid-state electrolytes due to its excellent lithium salt solubility [77]. Freitag et al. [78] tested the effects of SN on the electrochemical capabilities of electrospun PEO-based solid-state electrolytes. They found that the doped SN eliminated the crystalline regions of the solid-state electrolyte and largely improved their ionic conductivity. In addition, they optimized the concentration ratios of SN and LiBF_4_ multiple times. At a PEO:SN:LiBF_4_ ratio of 36:8:1, the ionic conductivity of the resulting solid-state electrolyte increased to 9 × 10^−4^ S/cm, which increased by an amplitude of 2 × 10^−4^ S/cm compared to that of other solid electrolytes without plasticizers.

In conclusion, the addition of the plasticizer can significantly enhance the ionic conductivity of electrospun PEO-based solid-state electrolytes. However, the introduction of plasticizers may also result in a decrease in their mechanical strength. Therefore, exploring a reasonable control of the doped plasticizers on the performance enhancement of electrospun PEO-based solid-state electrolytes is of significant importance.

#### 2.2.2. Inorganic Nanofillers

**Figure 8:**
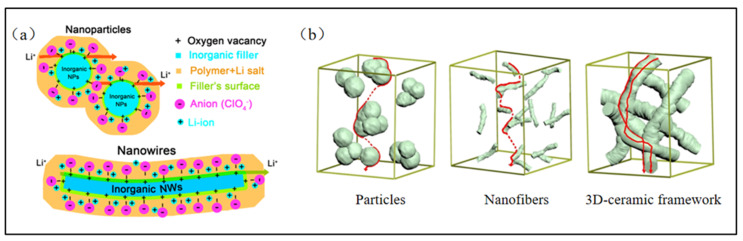
(**a**) Schematic illustration for Li-ion transport in the composite polymer electrolytes with nanoparticle and nanowire fillers. (Reprinted with permission from [83]. Copyright 2016, American Chemical Society.) (**b**) Schematics of conducting pathways for three geometrical structures filled in composite electrolytes. (Reprinted with permission from [84]. Copyright 2019, American Chemical Society.).

**Figure 9:**
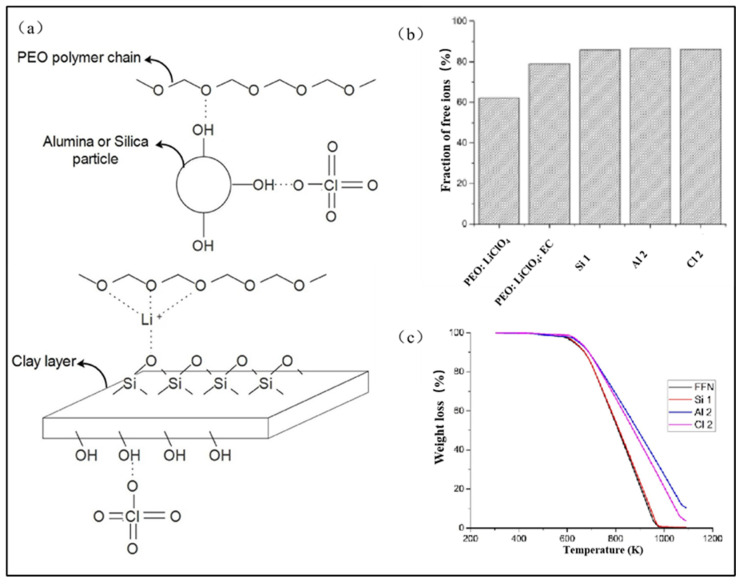
(**a**) Schematic illustration of the interaction between the fillers with PEO and lithium salt; (**b**) fraction of free ions of the as-spun electrolytes; (**c**) the TGA graph of the electrospun membrane containing three types of fillers. (Reprinted with permission from [86]. Copyright 2019, Springer Nature.).

Usually, inorganic nanofillers are used for enhancing the mechanical strength of electrospun PEO-based solid-state electrolytes, inhibiting the growth of lithium dendrites, triggering polymer chain segment restructuring, and reducing crystallinity, thereby improving ionic conductivity (Figure 8a) [79,80]. Additionally, inorganic nanofillers can facilitate the formation of new ion channels within the polymers, further optimizing the transfer of lithium ions (e.g., Figure 8b) [81]. Inert ceramic nanoparticles including titanium dioxide (TiO_2_), silicon dioxide (SiO_2_), aluminum oxide (Al_2_O_3_), magnesium oxide (MnO_2_), and zinc oxide (ZnO) are the most frequently utilized inorganic nanofillers [80]. The oxygen vacancy defects of these inert ceramic nanoparticles can be considered as Lewis acids, while the anions of the lithium salts and the ether oxygens of the PEO molecular chains are Lewis bases. Consequently, the mutual attractions between the ether bond of PEO and nanoparticles lessens the interactions between PEO molecular chains to some extent, effectively weakening the crystallinity of PEO materials (Figure 9a) [80,82]. This can strengthen the ionic conductivity of electrospun PEO-based solid-state electrolytes.

Zaccaria et al. [85] investigated the effects of inert ceramic nanoparticles on electrospun PEO micro/nanofibers by introducing SiO_2_ and tin dioxide (SnO_2_) to PEO during electrospinning. The experimental results indicated that the inclusion of nanoparticles improved the tensile strength of the electrospun fibers, suggesting a potential enhancement in the mechanical properties of electrospun PEO-based solid-state electrolytes. Banitaba et al. [86] compared the impacts of SiO_2_, Al_2_O_3_, and clay nanoparticles on the electrochemical properties of electrospun PEO-based solid-state electrolytes. Their findings demonstrated that the doped nanoparticles influence the electrical conductivity of the precursor solution, affecting the electrospinning process. A moderate amount of nanoparticles can effectively raise the fiber stretching rate and diminish the fiber diameter. However, an excessive amount of nanoparticles causes an increase in the solution viscosity, leading to the intertwining of polymer chains and the subsequent gradual increments in fiber diameters [87]. Additionally, Figure 9b,c illustrate that all three types of nanoparticles can effectively enhance the ionic conductivity and thermal stability of the resulting electrospun PEO-based solid-state electrolyte, and a suitable concentration of nanoparticles can improve the material’s mechanical properties. However, if the concentration of nanoparticles is excessively high, the surface energy causes agglomeration of the nanoparticles within the electrospun micro/nanofibers. This leads to stress concentration in the fibers, which induces increasing resistance for the migration of lithium ions, ultimately diminishing the mechanical and electrochemical properties of the electrospun PEO-based solid-state electrolyte [88].

Another study by Banitaba et al. [89] employed ZnO and TiO_2_ as nanoparticle fillers to fabricate electrospun PEO-based solid-state electrolytes, which exhibited superior mechanical properties and cycling stabilities. In some non-electrospun solid-state electrolytes, the doping of graphene improved their usability for practical applications. Based on this study, Abdollahi et al. [90] applied electrospinning to prepare solid-state electrolytes by combining graphene with PEO. They observed that the addition of graphene altered the conductivity of the electrospun solution, decreased the fiber diameter, and increased the internal porosity of the obtained solid-state electrolyte. Moreover, the interaction of graphene with PEO polymer chains reduced the material’s crystallinity and raised the electrical conductivity of the solid-state electrolyte.

**Figure 10:**
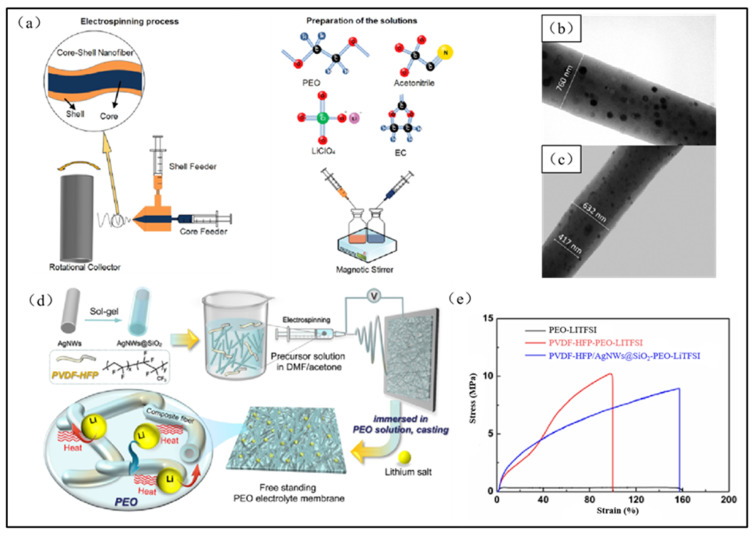
(**a**) Schematic illustrations of the polymer solution preparation and the electrospinning procedure; (**b**) TEM images of the PEO/EC/LiClO_4_ nanofiber loaded by SiO_2_ nanoparticles; (**c**) TEM images of electrospun core-shell nanofibers; (**d**) Schematic illustration of AgNWs@SiO_2_ and PVDF-HFP/AgNWs@SiO*2*/PEO/LiTFSI SPEs, (**e**) Stress-strain curves of SPEs (Reprinted with permission from [86]. Copyright 2021, Wiley).

The incorporation of multi-walled carbon nanotubes (MWCNTs) into electrospun PEO-based solid-state electrolytes has been found to enhance their cycling stability [91]. Compared to spherical nanoparticles, the elongated and tubular shape of MWCNTs affords larger contact areas, which is more advantageous for facilitating lithium-ion transport. Banitaba et al. [92] integrated MWCNT and SiO_2_ into the core of electrospun PEO micro/nanofibers using a coaxial electrospinning method and subsequently incorporated SiO_2_ into their shells (Figure 10a). This addition of MWCNT not only enhanced the cycling stability and the ionic conductivity of the resultant solid electrolyte but also decreased the diameters of the electrospun micro/nanofibers, improved lithium-ion mobility, and increased the ionic conductivity to 2.1 × 10^−4^ S/cm. Additionally, Gan et al. [93] investigated electrospun polyvinylidene fluoride–hexafluoropropylene copolymer (PVDF–HFP) micro/nanofibers containing silicon-coated silver nanotube fillers (AgNWs@SiO_2_) and prepared PEO-based solid-state electrolytes via casting method (Figure 10g). These AgNWs@SiO_2_-doped electrospun PVDF–HFP nanofibers endowed PEO-based solid-state electrolytes with superior mechanical and electrochemical properties. The resulting product not only displayed excellent charging and discharging rates but also a superior electrical cycling stability and improved ionic conductivity.

**Figure 11:**
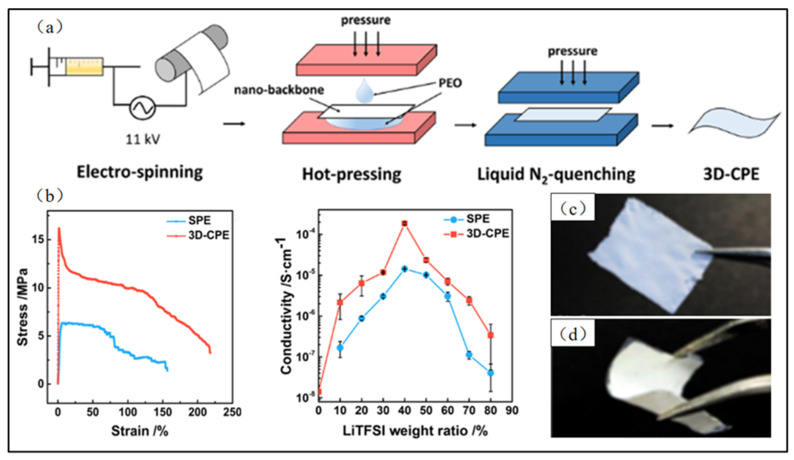
(**a**) Schematic picture of synthetic processes of 3D-CPEs; (**b**) stress–strain curves and room temperature conductivity as a function of LiTFSI concentration for SPE and 3D-CPEs; (**c**,**d**) as-calcined LLTO nano-backbone and a 3D-CPE. (Reprinted with permission from [95]. Copyright 2018, American Chemical Society.).

Nanowire fillers, as compared to nanoparticles, can form a large number of continuous ion transport channels in the solid-state electrolyte, thus increasing the migration rate of lithium ions and achieving a higher ionic conductivity [79]. In contrast to the previously mentioned direct electrospinning of PEO precursor solutions containing nanofillers to create micro/nanofibers, this type of solid-state electrolyte involves the fabrication of nanowire skeletons through electrospinning, followed by coating PEO on their surfaces using a solution casting method. Zhu et al. [94] prepared micro/nano skeletons doped with Li_0.33_La_0.557_TiO_3_ (LLTO) nanowire fillers via electrospinning and then cast PEO solutions onto them to create PEO-based solid-state electrolytes. When the content of the LLTO nanowire filler was 5 wt%, the ionic conductivities at room temperature and 60 ℃ increased to 5.53 × 10^−5^ S/cm and 3.63 × 10^−4^ S/cm, respectively. Such fillers can also increase the electrochemical stability of the prepared solid-state electrolyte whose electrochemical window achieve a value of 4.75 V. Additionally, Wang et al. [95] also prepared solid-state electrolytes of PEO doped with LLTO by electrospinning and a solution casting methodology, further enhancing their connections via a hot pressing method (Figure 11a). This approach resulted in solid-state electrolytes exhibiting excellent mechanical strength, superior thermal stability, and awesome electrochemical properties, as shown in Figure 11b-d. Zhang et al. [96] employed a similar method to prepare electrospun PEO-based solid-state electrolytes containing Li_7_La_3_Zr_2_O_12_ (LLZO) nanowire fillers. With an LLZO nanowire filler of 10 wt%, the ionic conductivities of the resulting solid electrolyte reached 9.87 × 10^−5^ S/cm and 8.66 × 10^−4^ S/cm at 30 °C and 60 °C, respectively. Notably, these electrolytes maintained a relatively stable voltage and exhibited adequate discharge-specific capacity even after several cycles.

Thus far, nanomaterials used in electrospun PEO-based solid-state electrolytes can be primarily classified into three categories: nanoparticles, nanotubes, and nanowire frameworks, as shown in Table 1. All three types of fillers can enhance the electrochemical performance and mechanical properties. The impact of various nanofillers on the ionic conductivity and tensile performance of electrospun PEO-based solid-state electrolytes are presented. In summary, the judicious addition of nanofillers helps improve the mechanical and electrochemical properties of the obtained electrolytes. Among the various nanofillers, the nanowire frameworks exhibit the most significant optimization of PEO-based solid-state electrolyte performance.

#### 2.2.3. Blending of PEO with Other Polymers

The third additive for enhancing the performance of electrospun PEO-based solid-state electrolytes involves blending PEO with other polymers. This approach allows leveraging the advantageous properties of both materials while mitigating their inherent disadvantages. Notably, this method can decrease PEO crystallinity, thereby enhancing the ionic conductivity of electrospun PEO-based solid-state electrolytes. The preparation process for precursor solutions of polymer blends is straightforward, and their composition can be easily adjusted, thus offering a greater convenience relative to other methods [59].

PVDF owns an excellent chemical stability and a high dielectric constant [102], which is biocompatible with PEO, making it a frequent choice for co-blending with PEO during electrospinning to enhance the performance of PEO-based solid-state electrolytes [103,104]. Monaca et al. [105] employed PEO of different molecular weights in combination with PVDF for electrospinning. They found that lower molecular weights of PEO resulted in electrospun micro/nanofibers of smaller diameters and uniform sizes, which showed a desirable electrochemical stability. Furthermore, the molecular chain orientation of PVDF in the finer electrospun nanofibers was higher, which was found to enhance the mechanical properties of the nanofiber membrane, resulting in excellent tensile strength of the solid-state electrolyte [106]. Li et al. [104] also co-blended PVDF and PEO for electrospinning. They noted strong polar forces between the functional groups of the two materials, which obviously improved the mechanical properties of the resulting solid-state electrolytes. Furthermore, Xing et al. [107] suggested that the blending process can reduce the crystallinity of the materials to a degree, leading to improved electrochemical properties. When the blending ratio of PEO:PVDF was 1:5, the resultant solid-state electrolyte demonstrated an ionic conductivity of 4.8 × 10^−3^ S/cm and an electrochemical window of 4.8 V at 303 K as well as superior specific capacity and coulombic efficiency. Prasanth et al. [103] prepared electrospun PEO-based solid-state electrolytes with PVDF, PEO, and a blend of both. The electrochemical performance of the samples produced through co-blending of PVDF and PEO was found to be remarkably superior to that of the single-component PVDF-based or PEO-based solid-state electrolytes.

**Figure 12:**
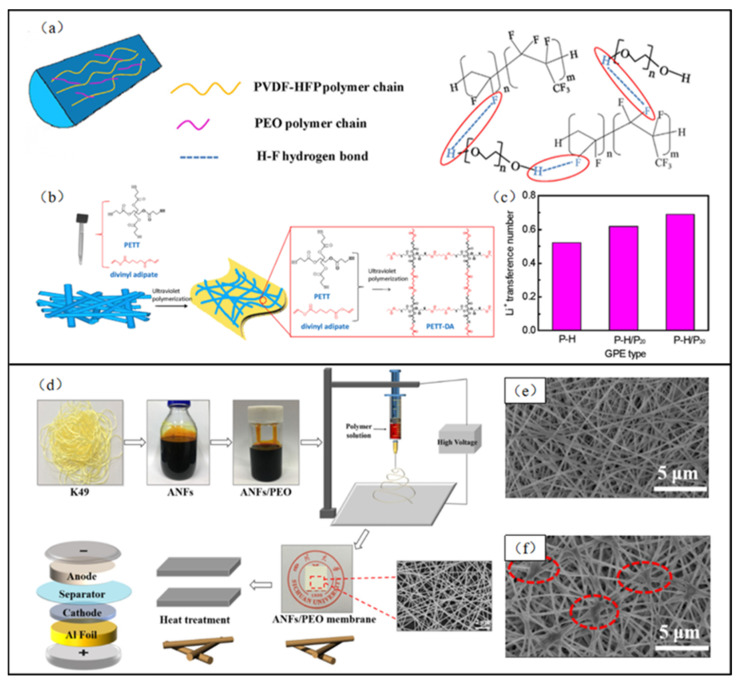
(**a**) Molecular chain model within fibers and hydrogen bonds between PVDF–HFP and PEO within fibers; (**b**) the formation process of the outside PETT–DA layer and ultraviolet polymerization between PETT and divinyl adipate; (**c**) lithium-ion transference numbers in three different content PEO electrospun fiber membrane. (Reprinted with permission from [108]. Copyright 2019 Elsevier Ltd.) (**d**) Schematic illustration of the preparation process of ANFs/PEO separators; (**e**,**f**) SEM images of electrospun fiber membranes before and after heat treatment. (Reprinted with permission from [110]. Copyright 2022, Elsevier Ltd.).

In addition to the prevalent blending of PVDF with PEO, the performance of PEO-based solid-state electrolytes can also be optimized by blending PEO with some polymers with complex molecular chains. Internal hydrogen bonds are formed when PEO is blended with PVDF–HPF, leading to increased intermolecular force between the polymers and reduced interaction with the solvent (as in Figure 12a). This can decrease the viscosity of the solution, accelerate solvent evaporation, reduce the diameters of electrospun micro/nanofibers, increase the porosity of the fiber membrane, and improve the electrochemical properties of the solid-state electrolyte. Wang et al. [108] blended PEO and PVDF–HPF for electrospinning and subsequently covered the surface of the electrospun fiber with ultraviolet-polymerized pentaerythritol tetraacetate and divinyl adipate (PETT–DA) polymer (Figure 12b). Although the formed hydrogen bonding between PEO and PVDF-HPF decreased the solution’s viscosity, an extremely low viscosity can lead to inhomogeneity in the thickness of the electrospun micro/nanofibers and compromise the mechanical properties of the resulting solid-state electrolytes. By adjusting the PEO concentration to 30%, this electrospun PEO-based solid-state electrolyte demonstrated an ionic conductivity of up to 8.71 × 10^−4^ S/cm while maintaining superior mechanical properties (Figure 12c).

In addition to PVDF and its copolymers, aramid nanofibers (ANFs) are considered an alternative polymer matrix for PEO-based solid electrolyte due to their exceptional mechanical properties, thermal stability, and safety [109]. Tang et al. [110] prepared precursor solutions by dissolving ANFs and co-blending them with PEO and electrospun them to obtain PEO-based solid-state electrolytes (Figure 12d). Due to weak interactions between PEO and ANFs, the electrospun fibers were further treated through a hot pressing to induce cross-linking sites within the fibers to enhance the material’s mechanical properties (Figure 12f). Test results demonstrated that the heat-treated electrospun fibers could limit the PEO recrystallization process and reduce internal resistance, resulting in an ionic conductivity of up to 4.33 × 10^−3^ S/cm.

**Figure 13:**
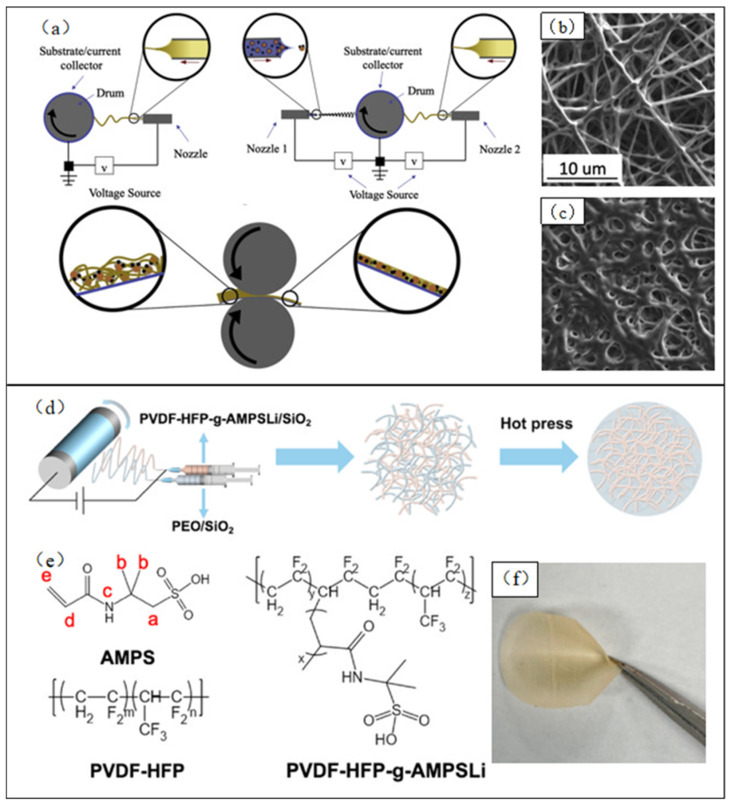
(**a**) Overview of manufacturing all-solid-state lithium-ion battery by using a synchronized electrospinning and electrospraying technique; (**b**) SEM image of electrospun porous mat made from PEO–LiClO_4_ fibers; (**c**) SEM image after partial compression; (**d**) schematic diagram of multi-nozzle electrospinning technology and hot pressing process. (Reprinted with permission from [111]. Copyright, 2019 Elsevier B.V.) (**e**) AMPS, PVDF–HFP, and AMPS–PVDF–HFP–AMPSLi molecular structure schematic diagram; (**f**) optical topography of PAS/PS with nozzle ratio (PVDF–HFP–g-AMPSLi:PEO) of 2:1. (Reprinted with permission from [112]. Copyright 2021, Elsevier Ltd.).

Additionally, surface coverage of electrospun PEO fibers with other polymers also presents a viable strategy for improving the performance of PEO-based solid-state electrolytes. Hafner et al. [111] applied a mixture of PVDF and Sn nanoparticles onto the surface of electrospun PEO nanofibers by electrostatic spraying, subsequently bonding them tightly by calendaring (Figure 13a). The SEM image is shown in Figure 13b,c; the resulting material formed an extremely robust internal mesh structure, conferring the material with superior mechanical properties and a high ionic conductivity. Furthermore, Hu et al. [112] utilized multi-needle electrospinning to combine PEO with other polymers (Figure 13d). They first grafted lithiated 2-acrylamido-2-methylpropanesulfonic acid (AMPSLi) onto the PVDF–HFP backbone to synthesize PVDF–HFP–g-AMPSLi (Figure 13e). Then, they added hydrophilic and hydrophobic SiO_2_ nanoparticles to the PEO and PVDF–HFP–g-AMPSLi solution to obtain two electrospinning precursor solutions, respectively. The two electrospun nanofibers displayed a stable three-dimensional network structure after a hot pressing. These nanofibers demonstrated ionic conductivity and electrochemical windows of 5.4 × 10^−5^ S/cm and 3.8 V at 60 °C, respectively. Moreover, they exhibited a superior multiplicative performance and a remarkably stable cycling performance in battery tests.

Efficient electrospun PEO-based solid-state electrolytes can also be fabricated by applying PEO as an auxiliary material onto other electrospun polymer fibers via casting. For instance, Wang et al. [113] produced polyimide (PI) nanofiber membranes through electrospinning and subsequently developed solid-state electrolytes by casting PEO solutions onto these membranes. This material exhibited appropriate mechanical properties, a high electrochemical window, adequate cycling stability, and an ionic conductivity reaching 4.2 × 10^−5^ S/cm at 60 °C.

PAN allows the cell to function at elevated temperatures and thus serves as an effective material of solid-state electrolyte due to its outstanding thermal stability. Ma et al. [114] soaked electrospun PAN fibers in PEO/LiTFSI solution and subsequently fabricated a solid-state electrolyte with superior mechanical properties via extension pressing of the composite. The covering of PEO and LiTFSI on the PAN fiber surface raised the electrochemical properties of the solid-state electrolytes, endowing them with extraordinary performance.

Thermoplastic polyurethane (TPU), known for its excellent elastomeric properties, is often selected as a matrix material of the solid-state electrolyte due to its excellent mechanical features [115]. However, due to TPU’s low ionic conductivity at room temperature, it necessitates a combination with other materials to break its limitations. Gao et al. [116] prepared PEO-based solid-state electrolytes by casting PEO onto electrospun TPU nanofiber membranes. The synergy of TPU fiber and PEO endowed this material with robust mechanical properties and superior ionic conductivity. Remarkably, it could reach an ionic conductivity of 6.1 × 10^−4^ S/cm at a temperature of 60 °C while exhibiting adequate stability in electrical cycling tests.

Hydrogen-bonding interactions between the amide bond on the poly(m-phenylene isophthalamide) (PMIA) benzene ring, PEO molecular chain, and lithium salt anion can inhibit PEO crystallization processes and promote LiTFSI dissociation, thereby enhancing ionic conductivity of the resulting solid-state electrolyte. Moreover, hydrogen bonding between PMIA and PEO can lead to the formation of a three-dimensional network structure in the solid-state electrolyte, improving its mechanical properties and thermal stability [117]. Gao et al. [118] uniformly sprayed PEO onto electrospun PMIA fibers through an electrostatic spraying approach. Then, they firmly bonded the two materials through calendering to prepare a solid-state electrolyte. This resultant sample exhibited a high fracture strength of 10.4 MPa and ionic conductivity of 2.9 × 10^−4^ S/cm at 30 °C.

In brief, this section mainly involves blending PEO with other polymer materials endowing robust mechanical properties, such as PVDF, aiming to enhance the mechanical features of electrospun PEO-based solid-state electrolytes. Furthermore, due to potential interactions between the distinct polymer materials, controllable adjustments in their proportions can effectively decrease the overall crystallinity, thereby boosting the ionic conductivity of the solid electrolyte.

### 2.3. Modification of PEO Molecular Structure

An alternative method to enhance the performance of electrospun PEO-based solid-state electrolytes involves the modification of PEO molecular chains. This avenue primarily relates to the formation of copolymers from PEO monomers and one or more additional monomers through polymerization reactions. This includes the production of alternating copolymers, random copolymers, block copolymers [119], and graft copolymers [120]. However, the fabrication process for such materials is generally intricate and costly. Consequently, there are limited studies concerning the preparation of electrospun PEO-based solid-state electrolytes for lithium-ion batteries in terms of these materials.

**Figure 14:**
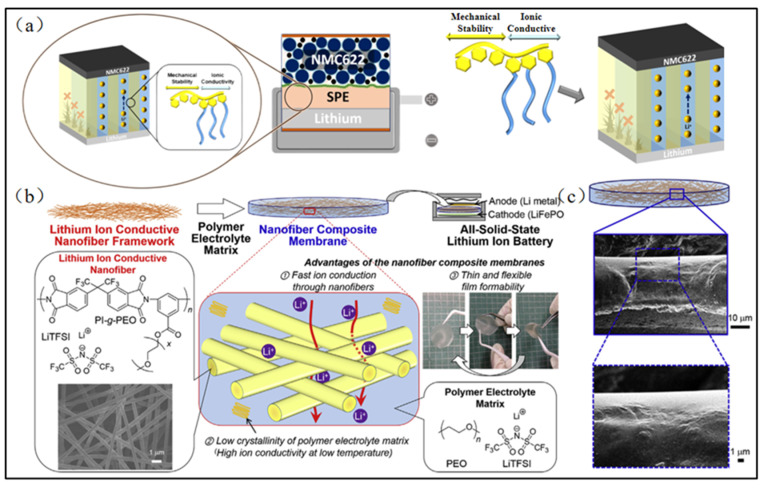
(**a**) Schematics of dendrite suppression by microphase separation of block copolymers. (Reprinted with permission from [121]. Copyright 2021, American Chemical Society.) (**b**) Schematic diagram of the process for preparing polymer composite film with lithium-ion conducting nanofiber framework from PI–g-PEO; (**c**) typical cross-sectional SEM images of the nanofiber composite membrane. (Reprinted with permission from [122]. Copyright 2019, Elsevier B.V.).

Block polymers are macromolecules comprising two or more chemically distinct polymers joined by covalent bonds. Appropriate selections of different polymers with varied molecular weights can yield a multitude of tailor-made materials with specific physicochemical properties. Despite the extensive applications of block copolymers in various fields, their utilization in electrospun PEO-based solid-state electrolytes for lithium-ion batteries remains in the nascent stages. Block copolymers composed of PEO and polystyrene (PS) demonstrate favorable microstructures when they are combined with lithium salts [121], making them a popular choice for PEO-based solid-state electrolytes. Lithium salts can be solvated in the PEO block, thereby providing an effective ion transport pathway for the resulting solid-state electrolytes. Simultaneously, PS can confer excellent mechanical strength to the material, thereby inhibiting lithium dendrite growth (Figure 14a). Chen et al. [122] prepared a PEO-based solid-state electrolyte by blending a synthesized PS–PEO–PS triblock copolymer with PVDF via electrospinning. According to the experimental results, the incorporation of PS–PEO–PS enhanced the thermal stability, reduced the internal crystallinity, and improved the electrochemical properties of the obtained product. The solid-state electrolytes with a concentration of PS–PEO–PS of 5% showed the lowest crystallinity, while their ionic conductivity reached 6.52 × 10^−4^ S/cm.

Watanabe et al. [123] fabricated PI–g-PEO graft copolymers by introducing the PEO side chain into the main chain of PI via an esterification reaction. Subsequently, they prepared them into nanofibers through electrospinning, which served as a solid-state electrolyte backbone. Finally, PEO and lithium salt were cast onto the electrospun nanofibrous backbone by the template casting method to prepare the electrospun PEO-based solid-state electrolytes (Figure 14b). They exhibited high mechanical strength, favorable electrochemical properties, high cycling stability, and a wide electrochemical window, demonstrating their potential applicability in lithium-ion batteries.

Copolymerizing PEO with other polymers also facilitates the preparation of PEO-based solid-state electrolytes with excellent properties. For instance, random copolymer PEO–co-PPO-based polymer electrolytes exhibit low crystallinity, superior mechanical properties, and adequate thermal stability. However, no scholarly work has yet reported the preparation of such materials into PEO-based solid-state electrolytes via electrospinning for lithium-ion batteries.

## 3. Conclusions and Prospect

With the rapid developments of wearable electronic products, flexible, compact, and high-energy-density batteries have recently attracted lots of attention. Lithium-ion batteries prepared with a solid-state electrolyte can maintain a high energy density while exhibiting a desirable safety performance, which could potentially be applied on a large scale for wearable electronic products in future.

Compared to other polymer materials, PEO is widely used in solid-state electrolytes due to its excellent lithium salt solubility and ionic conductivity. However, current PEO-based solid-state electrolytes face challenges such as poor mechanical properties and a narrow electrochemical window. Although they show good ionic conductivity, the developments of PEO-based solid-state electrolytes still lag behind that of traditional liquid electrolytes. To further advance the development of high-energy-density and ultra-safe lithium-ion batteries, this review discusses various strategies to enhance the electrochemical and mechanical performance of electrospun PEO-based solid-state electrolytes. In general, selecting suitable lithium salts with appropriate concentrations can improve the electrochemical performance of the obtained PEO-based solid-state electrolytes, although the impact is usually modest. Additionally, incorporating plasticizers into the PEO-based solid-state electrolyte can significantly improve its electrochemical ability but may compromise its mechanical properties and the safety. Therefore, current research efforts are primarily focused on enhancing their electrochemical and mechanical capabilities by incorporating inorganic nanoparticles or composing with other polymer materials. Inorganic nanofillers can enhance mechanical properties and improve the ion conduction by optimizing the transfer channels within electrospun micro/nanofibers. Similarly, blending electrospun PEO with other high-molecular-weight polymers not only reduces the crystallinity within the material but also combines their respective advantages to realize both high ionic conductivities and strong mechanical properties. And this preparation process has low production costs and an easy operation, facilitating a scale-up production. Finally, modification of PEO molecular chains can inhibit the growth of lithium dendrites and optimize the electrochemical performance abilities of the PEO-based solid-state electrolytes. Generally speaking, employing a multi-strategy approach can combine the strengths of different strategies and lead to further improvements in the performance of the resulting material. This allows for a comprehensive enhancement of various aspects of the material’s properties. This review aims to advance the future research on obtaining electrospun PEO-based solid-state electrolytes with superior performance for large-scale applications.

Although the aforementioned improvement strategies have contributed to the enhancement of the electrochemical and mechanical performance of electrospun PEO-based solid-state electrolytes, these electrolytes still encounter significant challenges in practical applications. For instance, significant changes of their ionic conductivity still exist as temperature varies, which seriously restricts its applications in lithium-ion batteries. Moreover, the technology of preparing PEO-based solid-state electrolytes by electrospinning is not yet mature, and there is still room for optimization of electrospinning process parameters. In addition, this technique is not yet feasible for large-scale industrialization. Therefore, the future optimization for electrospun PEO-based solid-state electrolytes primarily focuses on the following three aspects: (1) exploration of novel PEO-based materials is needed to simultaneously enhance ion conductivity and mechanical properties and reduce their sensitivity to temperature fluctuations. (2) optimization of the electrospun fiber’s morphology, diameter, arrangement, and other structures is needed to achieve a better ion transport efficiency and electrochemical performance; (3) realization of the industrial-scale production of electrospun PEO-based solid-state electrolyte is also necessary.

## Figures and Tables

**Table 1:** Effects of Filler Doping on Performance of Electrospun PEO-Based Solid-State Electrolytes.

Material	Avg. Fiber Diameter (μm)	Ionic Conductivity (S/cm)	Tensile Strength (MPa)	References
PEO SiO_2_	0.42 ± 0.08	-	1.7	[85]
PEO SnO_2_	0.37 ± 0.06	-	1.4	[85]
SiO_2_ LiClO_4_ PEO EC	0.99 ± 0.27	8.7 × 10^−5^	-	[86]
Al_2_O_3_ LiClO_4_ PEO EC	0.47 ± 0.11	5.9 × 10^−5^	-	[86]
LiClO_4_ PEO EC	0.83 ± 0.45	1.6 × 10^−5^	-	[86]
TiO_2_ LiClO_4_ PEO EC	0.936	8.5 × 10^−5^	0.2	[97]
SiO_2_ LiClO_4_ PEO	-	4.8 × 10^−5^	2.03	[98]
SiO_2_ LiFTSI PEO	-	1 × 10^−4^(30 °C)	3.1	[99]
PAN PEO GO LiI PC DEC	0.147 ± 0.023	1 × 10^−2^	-	[90]
SiO_2_ LiClO_4_ PEO MWCNT EC	-	2.1 × 10^−4^	-	[92]
AgNWs@SiO_2_ PVDF-HFP PEO LiFTSI	-	3.84 × 10^−5^(50 °C)	10.23	[93]
TiO_2_ LiClO_4_ PEO EC	0.56 ± 0.15	4.5 × 10^−5^	0.2	[89]
ZnO LiClO_4_ PEO EC	0.73 ± 0.22	3.5 × 10^−5^	0.1	[89]
Keggin-type hetero polyoxometalate LiClO_4_ PEO EC	0.607 ± 0.19	2.8 × 10^−4^	0.15	[100]
LLTO PEO LiFTSI	-	5.53 × 10^−5^	-	[94]
LLTO PEO LiFTSI	-	1.84 × 10^−4^	16.18	[95]
LLZAO PEO LiFTSI	-	9.87 × 10^−5^(30 °C)	-	[96]
LLTO PEO PVDF LiFTSI	0.088	6.02 × 10^−5^(50 °C)	6	[101]

## Data Availability

Not applicable.

## References

[1] Lu W., Yuan Z., Zhao Y., Zhang H., Zhang H., Li X. (2017). Porous membranes in secondary battery technologies. Chem. Soc. Rev..

[2] Hsu C.-Y., Liu R.-J., Hsu C.-H., Kuo P.-L. (2016). High thermal and electrochemical stability of PVDF-graft-PAN copolymer hybrid PEO membrane for safety reinforced lithium-ion battery. RSC Adv..

[3] Zhou G., Li F., Cheng H.-M. (2014). Progress in flexible lithium batteries and future prospects. Energy Environ. Sci..

[4] Baek S.-W., Honma I., Kim J., Rangappa D. (2017). Solidified inorganic-organic hybrid electrolyte for all solid state flexible lithium battery. J. Power Sources.

[5] Li Y.-H., Wu X.-L., Kim J.-H., Xin S., Su J., Yan Y., Lee J.-S., Guo Y.-G. (2013). A novel polymer electrolyte with improved high-temperature-tolerance up to 170 °C for high-temperature lithium-ion batteries. J. Power Sources.

[6] Scrosati B., Hassoun J., Sun Y.-K. (2011). Lithium-ion batteries. A look into the future. Energy Environ. Environ. Sci..

[7] Goodenough J.B., Park K.S. (2013). The Li-ion rechargeable battery: A perspective. J. Am. Chem. Soc..

[8] Liang J., Luo J., Sun Q., Yang X., Li R., Sun X. (2019). Recent progress on solid-state hybrid electrolytes for solid-state lithium batteries. Energy Storage Mater..

[9] Kim J.G., Son B., Mukherjee S., Schuppert N., Bates A., Kwon O., Choi M.J., Chung H.Y., Park S. (2015). A review of lithium and non-lithium based solid state batteries. J. Power Sources.

[10] Wu Y., Li Y., Wang Y., Liu Q., Chen Q., Chen M. (2022). Advances and prospects of PVDF based polymer electrolytes. J. Energy Chem..

[11] Yuriar-Arredondo K., Armstrong M.R., Shan B., Zeng W., Xu W., Jiang H., Mu B. (2018). Nanofiber-based Matrimid organogel membranes for battery separator. J. Membr. Sci..

[12] Zhou W., Wang Z., Pu Y., Li Y., Xin S., Li X., Chen J., Goodenough J.B. (2019). Double-Layer Polymer Electrolyte for High-Voltage All-Solid-State Rechargeable Batteries. Adv. Mater..

[13] Li D., Chen L., Wang T., Fan L.Z. (2018). 3D Fiber-Network-Reinforced Bicontinuous Composite Solid Electrolyte for Dendrite-free Lithium Metal Batteries. ACS Appl. Mater. Interfaces.

[14] Rathika R., Suthanthiraraj S.A. (2016). Ionic Interactions and Dielectric Relaxation of PEO/PVDF-Mg[(CF3SO2)2N2)] Blend Electrolytes for Magnesium Ion Rechargeable Batteries. Macromol. Res..

[15] Hu J., Wang W., Zhou B., Feng Y., Xie X., Xue Z. (2019). Poly(ethylene oxide)-based composite polymer electrolytes embedding with ionic bond modified nanoparticles for all-solid-state lithium-ion battery. J. Membr. Sci..

[16] Tarascon J.-M., Armand M. (2001). Issues and challenges facing rechargeable lithium batteries. Nature.

[17] Masoud E.M. (2015). Citrated porous gel copolymer electrolyte composite for lithium ion batteries application: An investigation of ionic conduction in an optimized crystalline and porous structure. J. Alloys Compd..

[18] Ye Q., Liang H., Wang S., Cui C., Zeng C., Zhai T., Li H. (2022). Fabricating a PVDF skin for PEO-based SPE to stabilize the interface both at cathode and anode for Li-ion batteries. J. Energy Chem..

[19] Song J., Wang Y., Wan C.C. (1999). Review of gel-type polymer electrolytes for lithium-ion batteries. J. Power Sources.

[20] Dias F.B., Plomp L., Veldhuis J.B. (2000). Trends in polymer electrolytes for secondary lithium batteries. J. Power Sources.

[21] Zhu Y., Hu A., Tang Q., Zhang S., Deng W., Li Y., Liu Z., Fan B., Xiao K., Liu J. (2018). Compact-Nanobox Engineering of Transition Metal Oxides with Enhanced Initial Coulombic Efficiency for Lithium-Ion Battery Anodes. ACS Appl. Mater. Interfaces.

[22] Zheng C., Li L., Wang K., Wang C., Zhang J., Xia Y., Huang H., Liang C., Gan Y., He X. (2020). Interfacial Reactions in Inorganic All-Solid-State Lithium Batteries. Batter. Supercaps.

[23] Fenton D. (1973). Complexes of Alkali Metal Ions with Poly (etylene oxide). Polymer.

[24] Shi C., Yu M. (2023). Flexible solid-state lithium-sulfur batteries based on structural designs. Energy Storage Mater..

[25] Cheng S., Smith D.M., Li C.Y. (2014). How Does Nanoscale Crystalline Structure Affect Ion Transport in Solid Polymer Electrolytes?. Macromolecules.

[26] Tarascon J.-M., Gozdz A., Schmutz C., Shokoohi F., Warren P. (1996). Performance of Bellcore’s plastic rechargeable Li-ion batteries. Solid State Ion..

[27] Hooper A., North J.M. (1983). The fabrication and performance of all solid state polymer-based rechargeable lithium cells. Solid State Ion..

[28] Bannister D., Davies G., Ward I., McIntyre J. (1984). Ionic conductivities for poly (ethylene oxide) complexes with lithium salts of monobasic and dibasic acids and blends of poly (ethylene oxide) with lithium salts of anionic polymers. Polymer.

[29] Bates J., Dudney N., Gruzalski G., Zuhr R., Choudhury A., Luck C., Robertson J. (1992). Electrical properties of amorphous lithium electrolyte thin films. Solid State Ion..

[30] Clericuzio M., Parker Jr W., Soprani M., Andrei M. (1995). Ionic diffusivity and conductivity of plasticized polymer electrolytes: PMFG-NMR and complex impedance studies. Solid State Ion..

[31] Michot T., Nishimoto A., Watanabe M. (2000). Electrochemical properties of polymer gel electrolytes based on poly (vinylidene fluoride) copolymer and homopolymer. Electrochim. Acta.

[32] Quartarone E., Mustarelli P., Magistris A. (1998). PEO-based composite polymer electrolytes. Solid State Ion..

[33] Zhang X., Ji L., Toprakci O., Liang Y., Alcoutlabi M. (2011). Electrospun nanofiber-based anodes, cathodes, and separators for advanced lithium-ion batteries. Polym. Rev..

[34] Ogasawara T., Débart A., Holzapfel M., Novák P., Bruce P.G. (2006). Rechargeable Li_2_O_2_ electrode for lithium batteries. J. Am. Chem. Soc..

[35] Ji X., Nazar L.F. (2010). Advances in Li–S batteries. J. Mater. Chem..

[36] Huang W., Pan Q., Qi H., Li X., Tu Y., Li C.Y. (2017). Poly (butylene terephthalate)-b-poly (ethylene oxide) alternating multiblock copolymers: Synthesis and application in solid polymer electrolytes. Polymer.

[37] Chen Y., Shi Y., Liang Y., Dong H., Hao F., Wang A., Zhu Y., Cui X., Yao Y. (2019). Hyperbranched PEO-based hyperstar solid polymer electrolytes with simultaneous improvement of ion transport and mechanical strength. ACS Appl. Energy Mater..

[38] Liu X., Liang Q., Chen L., Tang J., Liu J., Tang M., Wang Z. (2023). PEO-Based Solid-State Electrolytes Reinforced by High Strength, Interconnected MOF Networks. ACS Appl. Energy Mater..

[39] Liu S., Liu W., Ba D., Zhao Y., Ye Y., Li Y., Liu J. (2023). Filler-integrated composite polymer electrolyte for solid-state lithium batteries. Adv. Mater..

[40] Keller M., Appetecchi G.B., Kim G.-T., Sharova V., Schneider M., Schuhmacher J., Roters A., Passerini S. (2017). Electrochemical performance of a solvent-free hybrid ceramic-polymer electrolyte based on Li_7_La_3_Zr_2_O_12_ in P(EO) 15 LiTFSI. J. Power Sources.

[41] Chen R.J., Zhang Y.B., Liu T., Xu B.Q., Lin Y.H., Nan C.W., Shen Y. (2017). Addressing the Interface Issues in All-Solid-State Bulk-Type Lithium Ion Battery via an All-Composite Approach. ACS Appl. Mater. Interfaces.

[42] Anton F. (1934). Process and Apparatus for Preparing Artificial Threads.

[43] Taylor G.I. (1964). Disintegration of water drops in an electric field. Proceedings of the Royal Society of London. Ser. A Math. Phys. Sci..

[44] Doshi J., Reneker D.H. (1995). Electrospinning process and applications of electrospun fibers. J. Electrost..

[45] Banitaba S.N., Semnani D., Karimi M., Heydari-Soureshjani E., Rezaei B., Ensafi A.A. (2021). A comparative analysis on the morphology and electrochemical performances of solution-casted and electrospun PEO-based electrolytes: The effect of fiber diameter and surface density. Electrochim. Acta.

[46] Robert Ilango P., Peng S. (2019). Electrospinning techniques for Li, Na and K-ion batteries. Curr. Opin. Electrochem..

[47] Kim H., Jeong G., Kim Y.U., Kim J.H., Park C.M., Sohn H.J. (2013). Metallic anodes for next generation secondary batteries. Chem. Soc. Rev..

[48] Aurbach D., Zinigrad E., Cohen Y., Teller H. (2002). A short review of failure mechanisms of lithium metal and lithiated graphite anodes in liquid electrolyte solutions. Solid State Ion..

[49] Xu K. (2014). Electrolytes and interphases in Li-ion batteries and beyond. Chem. Rev..

[50] Zhou D., Liu R., He Y.-B., Li F., Liu M., Li B., Yang Q.-H., Cai Q., Kang F. (2016). SiO2Hollow Nanosphere-Based Composite Solid Electrolyte for Lithium Metal Batteries to Suppress Lithium Dendrite Growth and Enhance Cycle Life. Adv. Energy Mater..

[51] Gong W., Zhang Z., Wei S., Ruan S., Shen C., Turng L.-S. (2020). Thermosensitive polyacrylonitrile/polyethylene oxide/polyacrylonitrile membrane separators for prompt and safer thermal lithium-ion battery shutdown. J. Electrochem. Soc..

[52] Feng S., Shi D., Liu F., Zheng L., Nie J., Feng W., Huang X., Armand M., Zhou Z. (2013). Single lithium-ion conducting polymer electrolytes based on poly[(4-styrenesulfonyl)(trifluoromethanesulfonyl)imide] anions. Electrochim. Acta.

[53] Fullerton-Shirey S.K., Maranas J.K. (2009). Effect of LiClO_4_ on the structure and mobility of PEO-based solid polymer electrolytes. Macromolecules.

[54] Marzantowicz M., Krok F., Dygas J.R., Florjańczyk Z., Zygadło-Monikowska E. (2008). The influence of phase segregation on properties of semicrystalline PEO:LiTFSI electrolytes. Solid State Ion..

[55] Wang S. (2007). Development of Solid Polymer Electrolytes of Polyurethane and Polyether-Modified Polysiloxane Blends with Lithium Salts.

[56] Haider A., Haider S., Kang I.-K. (2018). A comprehensive review summarizing the effect of electrospinning parameters and potential applications of nanofibers in biomedical and biotechnology. Arab. J. Chem..

[57] Yalcinkaya F., Yalcinkaya B., Jirsak O. (2015). Influence of Salts on Electrospinning of Aqueous and Nonaqueous Polymer Solutions. J. Nanomater..

[58] Vorrey S., Teeters D. (2003). Study of the ion conduction of polymer electrolytes confined in micro and nanopores. Electrochim. Acta.

[59] Xue Z., He D., Xie X. (2015). Poly(ethylene oxide)-based electrolytes for lithium-ion batteries. J. Mater. Chem. A.

[60] Banitaba S.N., Semnani D., Fakhrali A., Ebadi S.V., Heydari-Soureshjani E., Rezaei B., Ensafi A.A. (2020). Electrospun PEO nanofibrous membrane enable by LiCl, LiClO_4_, and LiTFSI salts: A versatile solvent-free electrolyte for lithium-ion battery application. Ionics.

[61] McLin M., Angell C. (1992). Frequency-dependent conductivity, relaxation times, and the conductivity/viscosity coupling problem, in polymer-electrolyte solutions: LiClO_4_ and NaCF_3_SO_3_ in PPO 4000. Solid State Ion..

[62] Chang C.-W., Lai W.-C. (2020). A strategy for preparing solid polymer electrolytes via the electrospinning process. J. Taiwan Inst. Chem. Eng..

[63] Walke P., Freitag K.M., Kirchhain H., Kaiser M., van Wüllen L., Nilges T. (2018). Electrospun Li(TFSI)@Polyethylene Oxide Membranes as Solid Electrolytes. Z. Für Anorg. Und Allg. Chem..

[64] Bandara L., Dissanayake M., Mellander B.-E. (1998). Ionic conductivity of plasticized (PEO)-LiCF_3_SO_3_ electrolytes. Electrochim. Acta.

[65] Kim Y.-T., Smotkin E.S. (2002). The effect of plasticizers on transport and electrochemical properties of PEO-based electrolytes for lithium rechargeable batteries. Solid State Ion..

[66] Nicotera I., Ranieri G.A., Terenzi M., Chadwick A.V., Webster M.I. (2002). A study of stability of plasticized PEO electrolytes. Solid State Ion..

[67] Qian X., Gu N., Cheng Z., Yang X., Wang E., Dong S. (2002). Plasticizer effect on the ionic conductivity of PEO-based polymer electrolyte. Mater. Chem. Phys..

[68] Fergus J.W. (2010). Ceramic and polymeric solid electrolytes for lithium-ion batteries. J. Power Sources.

[69] Srivastava S., Schaefer J.L., Yang Z., Tu Z., Archer L.A. (2014). 25th anniversary article: Polymer-particle composites: Phase stability and applications in electrochemical energy storage. Adv. Mater..

[70] Krawiec W., Scanlon L., Fellner J., Vaia R., Vasudevan S., Giannelis E. (1995). Polymer nanocomposites: A new strategy for synthesizing solid electrolytes for rechargeable lithium batteries. J. Power Sources.

[71] Jacob M., Prabaharan S., Radhakrishna S. (1997). Effect of PEO addition on the electrolytic and thermal properties of PVDF-LiClO_4_ polymer electrolytes. Solid State Ion..

[72] Michael M., Jacob M., Prabaharan S., Radhakrishna S. (1997). Enhanced lithium ion transport in PEO-based solid polymer electrolytes employing a novel class of plasticizers. Solid State Ion..

[73] Walker C.W., Salomon M. (1993). Improvement of ionic conductivity in plasticized PEO-based solid polymer electrolytes. J. Electrochem. Soc..

[74] Banitaba S.N., Semnani D., Rezaei B., Ensafi A.A. (2019). Morphology and electrochemical and mechanical properties of polyethylene-oxide-based nanofibrous electrolytes applicable in lithium ion batteries. Polym. Int..

[75] Li T., Balbuena P.B. (1999). Theoretical studies of lithium perchlorate in ethylene carbonate, propylene carbonate, and their mixtures. J. Electrochem. Soc..

[76] Banitaba S.N., Semnani D., Heydari-Soureshjani E., Rezaei B., Ensafi A.A. (2020). The effect of concentration and ratio of ethylene carbonate and propylene carbonate plasticizers on characteristics of the electrospun PEO-based electrolytes applicable in lithium-ion batteries. Solid State Ion..

[77] Voigt N., van Wüllen L. (2014). The effect of plastic-crystalline succinonitrile on the electrolyte system PEO:LiBF_4_: Insights from solid state NMR. Solid State Ion..

[78] Freitag K.M., Kirchhain H., Wullen L.V., Nilges T. (2017). Enhancement of Li Ion Conductivity by Electrospun Polymer Fibers and Direct Fabrication of Solvent-Free Separator Membranes for Li Ion Batteries. Inorg. Chem..

[79] Liu W., Lin D., Sun J., Zhou G., Cui Y. (2016). Improved Lithium Ionic Conductivity in Composite Polymer Electrolytes with Oxide-Ion Conducting Nanowires. ACS Nano.

[80] Zhang S.S. (2006). A review on electrolyte additives for lithium-ion batteries. J. Power Sources.

[81] Meziane R., Bonnet J.-P., Courty M., Djellab K., Armand M. (2011). Single-ion polymer electrolytes based on a delocalized polyanion for lithium batteries. Electrochim. Acta.

[82] Wimalaweera K., Seneviratne V., Dissanayake M. (2017). Effect of Al_2_O_3_ ceramic filler on thermal and transport properties of poly (ethylene oxide)-lithium perchlorate solid polymer electrolyte. Procedia Eng..

[83] Liu W., Liu N., Sun J., Hsu P.C., Li Y., Lee H.W., Cui Y. (2015). Ionic conductivity enhancement of polymer electrolytes with ceramic nanowire fillers. Nano Lett..

[84] Li Z., Sha W.X., Guo X. (2019). Three-Dimensional Garnet Framework-Reinforced Solid Composite Electrolytes with High Lithium-Ion Conductivity and Excellent Stability. ACS Appl. Mater. Interfaces.

[85] Zaccaria M., Gualandi C., Fabiani D., Focarete M.L., Croce F. (2012). Effect of Oxide Nanoparticles on Thermal and Mechanical Properties of Electrospun Separators for Lithium-Ion Batteries. J. Nanomater..

[86] Banitaba S.N., Semnani D., Heydari-Soureshjani E., Rezaei B., Ensafi A.A. (2019). Electrospun polyethylene oxide-based membranes incorporated with silicon dioxide, aluminum oxide and clay nanoparticles as flexible solvent-free electrolytes for lithium-ion batteries. Jom.

[87] Drew C., Wang X., Samuelson L.A., Kumar J. (2003). The effect of viscosity and filler on electrospun fiber morphology. J. Macromol. Sci. Part A.

[88] Pant H.R., Bajgai M.P., Nam K.T., Seo Y.A., Pandeya D.R., Hong S.T., Kim H.Y. (2011). Electrospun nylon-6 spider-net like nanofiber mat containing TiO(2) nanoparticles: A multifunctional nanocomposite textile material. J. Hazard. Mater..

[89] Banitaba S.N., Semnani D., Heydari-Soureshjani E., Rezaei B., Ensafi A.A. (2019). Effect of titanium dioxide and zinc oxide fillers on morphology, electrochemical and mechanical properties of the PEO-based nanofibers, applicable as an electrolyte for lithium-ion batteries. Mater. Res. Express.

[90] Abdollahi S., Sadadi H., Ehsani M., Aram E. (2021). Highly efficient polymer electrolyte based on electrospun PEO/PAN/single-layered graphene oxide. Ionics.

[91] Banitaba S.N., Semnani D., Heydari-Soureshjani E., Rezaei B., Ensafi A.A. (2019). Nanofibrous poly(ethylene oxide)-based structures incorporated with multi-walled carbon nanotube and graphene oxide as all-solid-state electrolytes for lithium ion batteries. Polym. Int..

[92] Banitaba S.N., Semnani D., Heydari-Soureshjani E., Rezaei B., Ensafi A.A. (2020). Electrospun core-shell nanofibers based on polyethylene oxide reinforced by multiwalled carbon nanotube and silicon dioxide nanofillers: A novel and effective solvent-free electrolyte for lithium ion batteries. Int. J. Energy Res..

[93] Gan H., Li S., Zhang Y., Wang J., Xue Z. (2021). Electrospun Composite Polymer Electrolyte Membrane Enabled with Silica-Coated Silver Nanowires. Eur. J. Inorg. Chem..

[94] Zhu L., Zhu P., Fang Q., Jing M., Shen X., Yang L. (2018). A novel solid PEO/LLTO-nanowires polymer composite electrolyte for solid-state lithium-ion battery. Electrochimica Acta.

[95] Wang X., Zhang Y., Zhang X., Liu T., Lin Y.H., Li L., Shen Y., Nan C.W. (2018). Lithium-Salt-Rich PEO/Li(0.3)La(0.557)TiO(3) Interpenetrating Composite Electrolyte with Three-Dimensional Ceramic Nano-Backbone for All-Solid-State Lithium-Ion Batteries. ACS Appl. Mater. Interfaces.

[96] Zhang Y., Feng W., Zhen Y., Zhao P., Wang X., Li L. (2020). Enhancement of cycling stability of all-solid-state lithium-ion batteries with composite polymer electrolytes incorporating Li_6.25_La_3_Zr_2_Al_0.25_O_12_ nanofibers. Ionics.

[97] Banitaba S.N., Semnani D., Rezaei B., Ensafi A.A. (2019). Evaluating the electrochemical properties of PEO-based nanofibrous electrolytes incorporated with TiO2nanofiller applicable in lithium-ion batteries. Polym. Adv. Technol..

[98] Zhang L., Li Y., Shi L., Yao R., Xia S., Wang Y., Yang Y. (2022). Electrospun Polyethylene Oxide (PEO)-Based Composite polymeric nanofiber electrolyte for Li-Metal Battery. J. Phys. Conf. Ser..

[99] Kimura K., Tominaga Y. (2017). Ionic Liquid-Containing Composite Poly(ethylene oxide) Electrolyte Reinforced by Electrospun Silica Nanofiber. J. Electrochem. Soc..

[100] Banitaba S.N., Semnani D., Heydari-Soureshjani E., Rezaei B., Ensafi A.A., Taghipour-Jahromi A. (2020). Novel electrospun polymer electrolytes incorporated with Keggin-type hetero polyoxometalate fillers as solvent-free electrolytes for lithium ion batteries. Polym. Int..

[101] Nourisabet T., Jamshidi Aval H., Shidpour R., Naji L. (2022). Fabrication of a PEO-PVDF blend based polymer composite electrolyte with extremely high ionic conductivity via the addition of LLTO nanowires. Solid State Ion..

[102] Ding Y., Zhang P., Long Z., Jiang Y., Xu F., Di W. (2008). Preparation of PVdF-based electrospun membranes and their application as separators. Sci. Technol. Adv. Mater..

[103] Prasanth R., Shubha N., Hng H.H., Srinivasan M. (2014). Effect of poly(ethylene oxide) on ionic conductivity and electrochemical properties of poly(vinylidenefluoride) based polymer gel electrolytes prepared by electrospinning for lithium ion batteries. J. Power Sources.

[104] Li W., Wu Y., Wang J., Huang D., Chen L., Yang G. (2015). Hybrid gel polymer electrolyte fabricated by electrospinning technology for polymer lithium-ion battery. Eur. Polym. J..

[105] La Monaca A., De Giorgio F., Focarete M.L., Fabiani D., Zaccaria M., Arbizzani C. (2017). Polyvinylidene Difluoride–Polyethyleneoxide Blends for Electrospun Separators in Li-Ion Batteries. J. Electrochem. Soc..

[106] Gao K., Hu X., Dai C., Yi T. (2006). Crystal structures of electrospun PVDF membranes and its separator application for rechargeable lithium metal cells. Mater. Sci. Eng. B.

[107] Xing Y., Wu Y., Wang H., Yang G., Li W., Xu L., Jiang X. (2014). Preparation of hybrid polymer based on polyurethane lithium salt and polyvinylidene fluoride as electrolyte for lithium-ion batteries. Electrochim. Acta.

[108] Wang X., Hao X., Hengjing Z., Xia X., Tu J. (2020). 3D ultraviolet polymerized electrolyte based on PEO modified PVDF-HFP electrospun membrane for high-performance lithium-sulfur batteries. Electrochim. Acta.

[109] Tung S.O., Fisher S.L., Kotov N.A., Thompson L.T. (2018). Nanoporous aramid nanofibre separators for nonaqueous redox flow batteries. Nat. Commun..

[110] Tang W., Liu Q., Luo N., Chen F., Fu Q. (2022). High safety and electrochemical performance electrospun para-aramid nanofiber composite separator for lithium-ion battery. Compos. Sci. Technol..

[111] Hafner S., Guthrey H., Lee S.-H., Ban C. (2019). Synchronized electrospinning and electrospraying technique for manufacturing of all-solid-state lithium-ion batteries. J. Power Sources.

[112] Hu T., Shen X., Peng L., Liu Y., Wang X., Ma H., Zhang P., Zhao J. (2021). Preparation of single-ion conductor solid polymer electrolyte by multi-nozzle electrospinning process for lithium-ion batteries. J. Phys. Chem. Solids.

[113] Wang Q., Yuan B., Lu Y., Shen F., Zhao B., Han X. (2021). Robust and high thermal-stable composite polymer electrolyte reinforced by PI nanofiber network. Nanotechnology.

[114] Ma Y., Wan J., Yang Y., Ye Y., Xiao X., Boyle D.T., Burke W., Huang Z., Chen H., Cui Y. (2022). Scalable, Ultrathin, and High-Temperature-Resistant Solid Polymer Electrolytes for Energy-Dense Lithium Metal Batteries. Adv. Energy Mater..

[115] Zhai Y., Xiao K., Yu J., Ding B. (2015). Fabrication of hierarchical structured SiO_2_/polyetherimide-polyurethane nanofibrous separators with high performance for lithium ion batteries. Electrochim. Acta.

[116] Gao M., Wang C., Zhu L., Cheng Q., Xu X., Xu G., Huang Y., Bao J. (2019). Composite polymer electrolytes based on electrospun thermoplastic polyurethane membrane and polyethylene oxide for all-solid-state lithium batteries. Polym. Int..

[117] Vashishta P., Mundy J.N., Shenoy G. Fast Ion Transport in Solids: Electrodes and Electrolytes. Proceedings of the International Conference on Fast Ion Transport in Solids, Electrodes, and Electrolytes.

[118] Gao L., Liang H., Li J., Cheng B., Deng N., Kang W. (2021). The high-strength and ultra-thin composite electrolyte using one-step electrospinning/electrostatic spraying process for interface control in all-solid-state lithium metal battery. J. Power Sources.

[119] Sun J., Stone G.M., Balsara N.P., Zuckermann R.N. (2012). Structure–Conductivity Relationship for Peptoid-Based PEO–Mimetic Polymer Electrolytes. Macromolecules.

[120] He D., Cho S.Y., Kim D.W., Lee C., Kang Y. (2012). Enhanced Ionic Conductivity of Semi-IPN Solid Polymer Electrolytes Based on Star-Shaped Oligo(ethyleneoxy)cyclotriphosphazenes. Macromolecules.

[121] Butzelaar A.J., Roring P., Mach T.P., Hoffmann M., Jeschull F., Wilhelm M., Winter M., Brunklaus G., Theato P. (2021). Styrene-Based Poly(ethylene oxide) Side-Chain Block Copolymers as Solid Polymer Electrolytes for High-Voltage Lithium-Metal Batteries. ACS Appl. Mater. Interfaces.

[122] Watanabe T., Inafune Y., Tanaka M., Mochizuki Y., Matsumoto F., Kawakami H. (2019). Development of all-solid-state battery based on lithium ion conductive polymer nanofiber framework. J. Power Sources.

[123] Chen Y.-n., Xiao Q., Li Q.-y., Ren S.-j. (2020). Preparation and characterization of electrospun crosslinked gel polymer electrolytes. Acta Polym. Sin..

